# Histamine Induces Alzheimer's Disease-Like Blood Brain Barrier Breach and Local Cellular Responses in Mouse Brain Organotypic Cultures

**DOI:** 10.1155/2015/937148

**Published:** 2015-11-30

**Authors:** Jonathan C. Sedeyn, Hao Wu, Reilly D. Hobbs, Eli C. Levin, Robert G. Nagele, Venkat Venkataraman

**Affiliations:** ^1^Graduate School of Biomedical Sciences, Rowan University, Stratford, NJ 08084, USA; ^2^Department of Cell Biology, Rowan School of Osteopathic Medicine, Stratford, NJ 08084, USA; ^3^Biomarker Discovery Center, New Jersey Institute for Successful Aging, Rowan University School of Osteopathic Medicine, Stratford, NJ 08084, USA; ^4^Department of Geriatrics and Gerontology, Rowan University School of Osteopathic Medicine, Stratford, NJ 08084, USA

## Abstract

Among the top ten causes of death in the United States, Alzheimer's disease (AD) is the only one that cannot be cured, prevented, or even slowed down at present. Significant efforts have been exerted in generating model systems to delineate the mechanism as well as establishing platforms for drug screening. In this study, a promising candidate model utilizing primary mouse brain organotypic (MBO) cultures is reported. For the first time, we have demonstrated that the MBO cultures exhibit increased blood brain barrier (BBB) permeability as shown by IgG leakage into the brain parenchyma, astrocyte activation as evidenced by increased expression of glial fibrillary acidic protein (GFAP), and neuronal damage-response as suggested by increased vimentin-positive neurons occur upon histamine treatment. Identical responses—a breakdown of the BBB, astrocyte activation, and neuronal expression of vimentin—were then demonstrated in brains from AD patients compared to age-matched controls, consistent with other reports. Thus, the histamine-treated MBO culture system may provide a valuable tool in combating AD.

## 1. Introduction

As the most common form of dementia, Alzheimer's disease (AD) is currently affecting over 5.5 million people in the United States and more than 35 million worldwide [1, 2]. The hallmark of the disease is progressive cognitive decline that results in loss of language/communication skills, difficulty in learning, loss of memory, and alterations in personality/mood [3–5]. The pathological changes seen in AD include synaptic loss, dendrite retraction, neuronal cell death, inflammation, astrocyte activation, blood-brain barrier (BBB) breakdown, and the accumulation of amyloid peptide 1–42 (A*β*42) within neurons and plaques throughout the hippocampus and cerebral cortex [6–12]. It has been noticed that breakdown of the BBB is a particularly important development in AD progression, as it allows for the leakage of damaging humoral elements into the brain parenchyma [7, 13, 14].

The BBB is comprised of specialized vascular endothelial cells that are connected to one another via tight junctions. These endothelial cells are different from those in other parts of the mammalian body in that they lack fenestrations and therefore do not allow for free exchange of solutes between the blood and the brain parenchyma [15, 16]. Additionally, astrocytic foot processes wrap around the blood vessels and play an important role in allowing endothelial cells to form and maintain their normally protective, tight seal [17]. When BBB breach occurs in the AD brains, it allows for the extravasation of blood-borne A*β*42, brain-reactive autoantibodies, and inflammatory factors into the normally immune-privileged brain parenchyma [18–20]. Access of the previously excluded and potentially damaging blood-borne plasma elements to the brain interstitium results in disruption of brain homeostasis, impaired neuronal function, and eventually neuronal loss [7, 21–23]. These deleterious effects on neurons are apparently buffered by activation of neuronal repair mechanisms, one of which involves neuronal expression of vimentin. Vimentin is an intermediate filament protein that is found primarily in endothelial cells and developing neurons [24, 25]. Vimentin expression in neurons has been linked temporally and spatially to dendrite repair in neurons of the cerebral cortex in AD and mouse brains subjected to traumatic injury [26]. Furthermore, injury or disease of the CNS, such as AD, causes gliosis, which is characterized by activation of astrocytes and an increased expression of glial fibrillary acidic protein (GFAP) in these cells [27–29]. Therefore, BBB breakdown has been assigned as a key event in initiating damage and damage responses in both neurons and glial cells in AD.

While the deleterious effects of BBB breakdown are well documented, its origin remains unclear. Several molecules have been implicated, such as bradykinin, nitric oxide, oxygen radicals, and histamine [16]. Among these, histamine is a proinflammatory mediator derived from the amino acid histidine [30]. It is present throughout the mammalian body, predominantly localized to mast cell granules and basophils. Histamine also acts as a neurotransmitter and is released by histaminergic neurons of the tuberomamillary nucleus of the posterior hypothalamus [31]. Upon injury or trauma, an inflammatory response occurs and causes the release of histamine [16, 32], which then induces the BBB breach [33]. Several* in vivo* studies have shown that histamine, whether applied luminally or abluminally to microvasculature of the brain, increases BBB permeability by opening the interendothelial cell tight junctions [33–37]. Moreover, histamine is also shown to induce a swelling of perivascular glial foot processes when applied luminally via carotid artery infusion [38, 39]. While histamine has been previously shown to induce BBB permeability* in vivo*, it is not yet known if it could cause a similar effect* in vitro* leading to generation of additional brain pathologies, for example, the neuronal and glial cell responses seen in neurodegenerative diseases such as AD.

We chose primary mouse brain organotypic (MBO) slice cultures as the model system, which has shown promise in assessing the response of brain cells to a wide variety of external stimuli [40, 41]. In this study, we report that histamine elicits responses in MBO cultures that include a breakdown of the BBB as shown by IgG leakage into the brain parenchyma, astrocyte activation as evidenced by increased expression of GFAP and a neuronal damage-response as suggested by increased vimentin expression in these cells. We demonstrate that these changes are also observed in the brains of AD patients, consistent with earlier reports. Therefore, we propose that the histamine-treated MBO slice culture system may provide an attractive option to analyze cellular changes and responses observed in AD patients as well as to screen potential drug candidates for AD.

## 2. Materials and Methods

### 2.1. Ethics Statement

All experiments were conducted in compliance with the NIH guidelines and in accordance with protocols approved by the Institutional Review Board and Institutional Animal Care and Use Committee at Rowan University, School of Osteopathic Medicine.

### 2.2. Antibodies

Human IgG antibodies (polyclonal, Cat. number BA-3000, diluted at 1 : 2000) and mouse IgG antibodies (polyclonal, Cat. number BA-9200, diluted at 1 : 2000) were obtained from Vector Laboratories (Foster City, CA). GFAP antibodies were obtained from Millipore (Billerica, MA; polyclonal, Cat. number AB5804, diluted at 1 : 1000). Vimentin antibodies were obtained from Sigma (Saint Louis, MO; monoclonal, Cat. number V6630, diluted at 1 : 200). The specificity of the antibodies has been demonstrated earlier [26].

### 2.3. Animals

C57BL/6J mice were obtained from Jackson Laboratories (Bar Harbor, ME) and used at 9 months of age. Mice were maintained on* ad libitum* food and water with 12-hour light/dark cycle in an AAALAC-accredited vivarium.

### 2.4. Primary Mouse Brain Organotypic Cultures

Primary mouse brain organotypic cultures were prepared as described previously [26]. Briefly, the brains were removed from C57BL/6 mice (*n* = 8) and cut to either 1 mm or 2 mm thickness coronally using a tissue chopper. The brain slices were then placed in medium containing 25% inactivated horse serum, 25% Hanks' BSS, 50% DMEM, and 25 mg/L penicillin-streptomycin (Invitrogen, Carlsbad, CA) in 6-well culture dishes and maintained for 30 minutes at room temperature. The brain slices were then moved to either fresh media (control) or fresh media containing 450 *μ*M histamine (Sigma, Saint Louis, MO, Cat. number H7125-1G) in 6-well culture dishes for 1 hour at 37°C in a 5% CO_2_-enriched atmosphere. The brain slices were processed for immunohistochemistry as described below.

The MBO slices were stored in 4% PFA overnight at 4°C and processed according to previously published protocols [42–44]. The brain slices were then infiltrated with 30% sucrose in PBS overnight at 4°C under constant, gentle agitation. Using a Leica cryostat, 12 *μ*m thick frozen sections were cut, mounted onto Fisher Super Frost Plus slides, and air dried. The slides were stored until use.

### 2.5. Human Brain Tissue

Brain tissue from patients with sporadic AD (*n* = 21) and age-matched, neurologically normal individuals (*n* = 13) were obtained from the Harvard Brain Tissue Resource Center (Belmont, MA), the Cooperative Human Tissue Network (Philadelphia, PA), the UCLA Tissue Resource Center (Los Angeles, CA), and Slidomics (Cherry Hill, NJ). Postmortem intervals were less than 24 hours and pathological confirmation of AD was evaluated according to the criteria defined by the National Institute on Aging and the Reagan Institute Working Group on Diagnostic Criteria for the Neuropathological Assessment of AD [45]. AD tissues displayed amyloid plaques and neurofibrillary tangles, and control tissues exhibited no gross pathology and minimal localized microscopic AD-like neuropathology. Tissues were processed for routine paraffin embedding and sectioning according to established protocols [46, 47].

### 2.6. Immunohistochemistry

Immunohistochemistry for the paraffin-embedded tissues was carried out as previously described [46, 47]. Briefly, tissues were deparaffinized using xylene and then rehydrated through a graded series of decreasing concentrations of ethanol. Next, protein antigenicity was enhanced by microwaving sections in citrate buffer. The paraffin-embedded tissues were then processed in the same way as the frozen sections described below.

Immunohistochemistry for the frozen sections was carried out as previously described [26, 42–44]. Briefly, tissues were rehydrated with PBS for 2 minutes. The endogenous peroxidase was quenched by treating sections with 0.3% H_2_O_2_ for 30 minutes. First, sections were incubated in blocking serum for 30 minutes and then treated with primary antibodies at appropriate dilutions for 1 hour at room temperature. Next, sections were thoroughly rinsed with PBS and incubated with biotin-labeled secondary antibody for 30 minutes at room temperature. Sections were then treated with the avidin-peroxidase-labeled biotin complex (ABC, Vector Labs, Foster City, CA) and visualized by treating with either 3-3-diaminobenzidine-4-HCL (DAB)/H_2_O_2_ (Biomeda, Foster City, CA) or NovaRed (Vector Labs, Foster City, CA, Cat. number SK-4800). Sections were then lightly counterstained with hematoxylin, dehydrated through increasing concentrations of ethanol, cleared in xylene, and mounted in Permount. Specimens were examined and photographed with a Nikon FXA microscope, and digital images were recorded using a Nikon DXM1200F digital camera and processed using Image Pro Plus (Phase 3 Imaging, Glen Mills, PA).

### 2.7. Quantitation and Statistics

Leaky and nonleaky vessels, GFAP-positive and GFAP-negative astrocytes, and vimentin-positive and vimentin-negative neurons were imaged in sections of MBO culture slices. Images were optimized and counting was performed using the counting feature of Adobe Photoshop. Three sections were examined per treatment group, with at least ten viewing fields counted from each section, for a total of 30 viewing fields per treatment group. Only blood vessels with endothelial cell nuclei, astrocytes with their nuclei, and neurons with their nuclei within the plane of section were included in the count. Blood vessels were considered leaky if they showed a gradient of immunostaining surrounding the vessel. Astrocytes were considered GFAP-positive if they showed staining within their cell body and dendrites. Neurons were considered vimentin-positive if they showed immunostaining in the cell body and/or main apical dendrite. The percentage of total leaky vessels, GFAP-positive astrocytes, and vimentin-positive neurons for histamine treated and control slices were plotted. Mean ± SEM was used to represent the variations within each group. A two-tailed Student's* t*-test or one-way ANOVA was performed to determine the statistical significance. ^*∗*^ *P* < 0.05; ^*∗∗*^ *P* < 0.01; ^*∗∗∗*^ *P* < 0.001.

## 3. Results

While the use of MBO cultures to study the brain's response to specific treatments and/or conditions is well-documented [26, 40, 41, 48], there is no information on the integrity of the blood vessels in MBO cultures, a critical determinant in these experiments. To fill this gap, MBO slice cultures were generated and treated with or without histamine as described in Section 2. To further verify the successful penetrance of histamine, 2 mm slices were sectioned and grouped at different depths from the slice surface (proximal, 0–350 *μ*m; middle, 350–700 *μ*m; and distal, 700–1050 *μ*m from the slice surface).

### 3.1. Histamine Treatment Increases Blood Vessel Permeability in MBO Cultures

**Figure 1:**
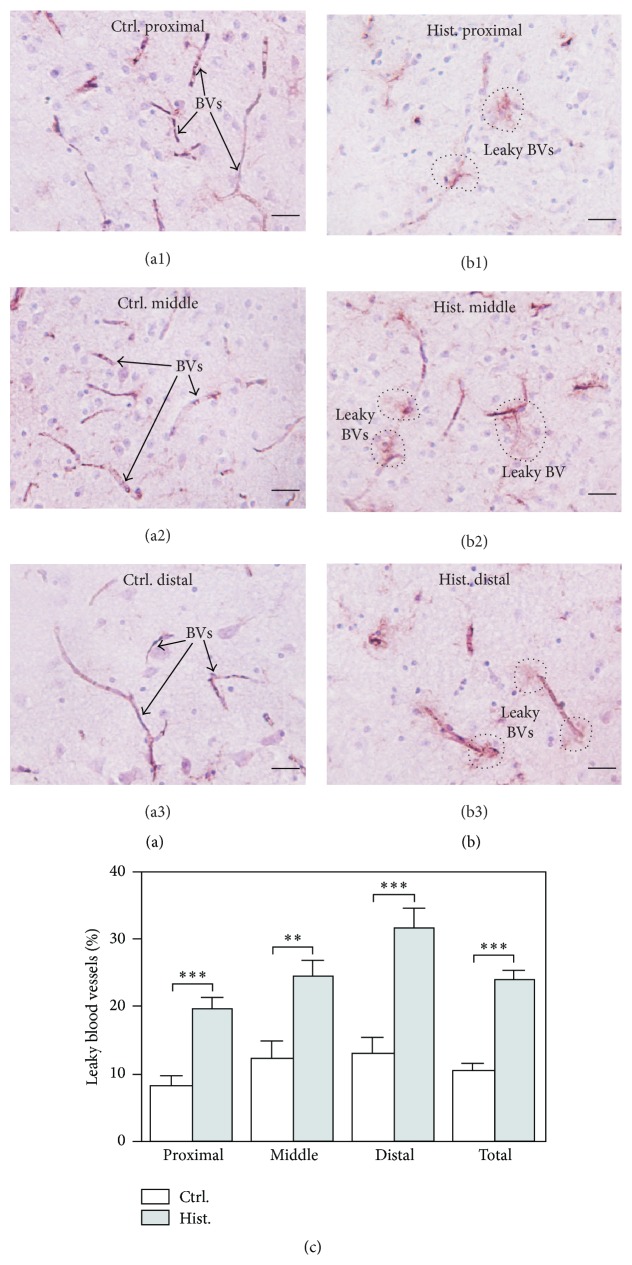
Histamine causes BBB breakdown in MBO cultures. ((a1)–(a3)) In control MBO sections, IgG (visualized by NovaRED) is mainly confined to the lumen in blood vessel (BV). Arrows indicate BVs. ((b1)–(b3)) In histamine-treated MBO sections, extravasated IgG (visualized by NovaRED) surrounding BVs is indicated by dotted circles. Sections (a1) and (b1) are from the proximal portion of the MBO tissue (0–350 *μ*m from the slice surface); sections (a2) and (b2) are from the middle portion of the MBO tissue (350–700 *μ*m from the slice surface); and sections (a3) and (b3) are from the distal portion of the MBO tissue (700–1050 *μ*m from the slice surface). The sections are counterstained with hematoxylin to show nucleus in purple as described in Section 2. Scale bars, 100 *μ*m. (c) Quantification shows more leaky BVs in the histamine-treated MBO cultures compared to the controls. Leaky BVs are counted from multiple images, normalized by the total numbers of all BVs and plotted. Mean ± SEM is used to represent the variations within each group. ^*∗*^ *P* < 0.05; ^*∗∗*^ *P* < 0.01; ^*∗∗∗*^ *P* < 0.001.

IgG localized in perivascular leak clouds emerging from a discrete region along the length of vessels have been identified as a marker for BBB breakdown [7, 49]. Conversely, perivascular leak clouds are rare and IgG is restricted to the lumen of blood vessels (BVs) under healthy status. To detect the extravasated IgG in the brain interstitium, antibodies against mouse IgG were used for immunostaining. Representative images from untreated controls (panels (a1), (a2), and (a3)) and histamine-treated samples (panels (b1), (b2), and (b3)) are presented in Figure 1. Quantification from multiple fields was also acquired and plotted in Figure 1(c).

The results demonstrate more perivascular leak clouds in the histamine-treated MBO cultures (indicated by dotted circles in Figure 1(b)) compared to the corresponding untreated controls (Figure 1(a)). In the histamine-treated 2 mm slices, the percentage of leaky blood vessels (BVs) increases significantly (*P* < 0.001) compared to controls (Figure 1(c)). Furthermore, the difference is significant regardless of the distance from the slice surface—proximal, middle, and distal (Figure 1(c)). An additional observation is that there is no significant difference in the percentage of leaky vessels detected among the three groups in control sections based on one-way ANOVA (*P* = 0.3076). On the other hand, in the histamine-treated samples, the three groups show differences in the percentage of leaky BVs (*P* = 0.0015, one-way ANOVA); especially, the sections distal from the slice surface exhibit significantly more leaky BVs compared to the proximal sections (*P* < 0.001, two-tailed student *t*-test). These results suggest that histamine is definitely able to exert its effect through the 2 mm thickness. At present, it is unclear whether the difference between the proximal and distal sections is biological or technical in origin. Overall, the results above demonstrate an increase of BV permeability upon histamine treatment in the MBO cultures.

### 3.2. Histamine Treatment Leads to Astrocyte Activation in MBO Cultures

**Figure 2:**
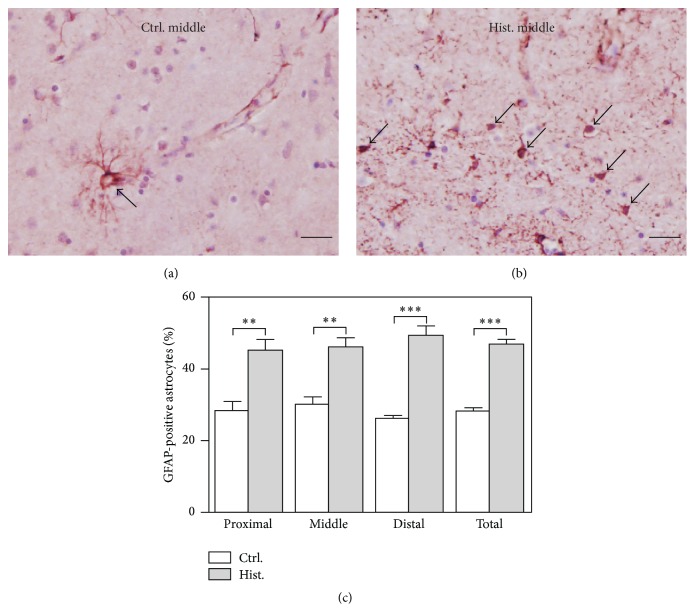
Histamine leads to astrocyte activation in MBO cultures. (a) Control MBO cultures show few astrocytes that are GFAP-positive (visualized by NovaRED and indicated by arrows). (b) Histamine-treated MBO cultures show many intensely GFAP-positive astrocytes (visualized by NovaRED and indicated by arrows). In both (a) and (b), sections are from the middle portion of the MBO tissue (350–700 *μ*m from the slice surface) and counterstained with hematoxylin to show nucleus in purple as described in Section 2. Scale bar, 100 *μ*m. (c) Quantification shows more GFAP-positive astrocytes in the histamine-treated MBO cultures compared to the controls. GFAP-positive astrocytes are counted from multiple images, normalized by the total cell numbers and plotted. Mean ± SEM is used to represent the variations within each group. ^*∗*^ *P* < 0.05; ^*∗∗*^ *P* < 0.01; ^*∗∗∗*^ *P* < 0.001.

To examine how histamine affects activation of astrocytes, changes in GFAP expression in MBO cultures were investigated by immunostaining with anti-GFAP antibodies. Quantitative analyses from all three groups (proximal, middle, and distal) are presented in Figure 2, along with images from the middle sections with or without histamine treatment as an example. Compared with the control (Figure 2(a)), an increase in the number of GFAP-positive cell bodies and a more scattered distribution of GFAP are observed in the histamine-treated samples (Figure 2(b)). Quantitative analyses also reveal significantly elevated density of GFAP-positive astrocytes upon histamine treatment compared to the controls (*P* < 0.001, Figure 2(c)). There is no significant difference among the three groups—proximal, middle, and distal (untreated, *P* = 0.5124; treated, *P* = 0.6343; one-way ANOVA), leading to the conclusion that the response is comparable regardless of the distance from the slice surface. Thus, the results here show that histamine treatment in MBO cultures elicits astrocyte responses, indicated by increased GFAP expression.

### 3.3. Vimentin Is Expressed in Neurons in Response to Exposure to Histamine

**Figure 3:**
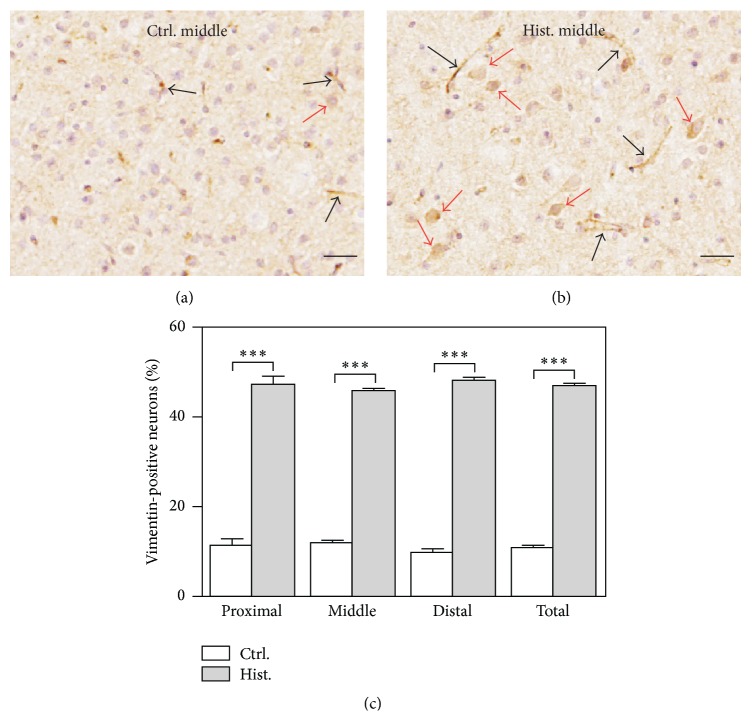
Histamine leads to neuronal damage and a concurrent damage response in MBO cultures. (a) In control MBO cultures, vimentin expression (visualized by DAB in brown) is found in BVs (indicated by black arrows) and very few neurons at a low level (indicated by red arrows). (b) In the histamine-treated MBO cultures, vimentin (visualized by DAB in brown) is localized in the neurons (indicated by red arrows) as well as BVs (indicated by black arrows). In both (a) and (b), sections are from the middle portion of the MBO tissue (350–700 *μ*m from the slice surface) and counterstained with hematoxylin to show nucleus in purple as described in Section 2. Scale bar, 100 *μ*m. (c) Quantification shows more vimentin-positive neurons in the histamine-treated MBO cultures compared to the controls. Vimentin-positive neurons are counted from multiple images, normalized by the total cell numbers, and plotted. Mean ± SEM is used to represent the variations within each group. ^*∗*^ *P* < 0.05; ^*∗∗*^ *P* < 0.01; ^*∗∗∗*^ *P* < 0.001.

Under normal, nonpathological conditions, vimentin is expressed in the brain only by endothelial cells and developing neurons. Our studies show that neurons can undergo a localized damage-response that includes the expression of vimentin to reestablish their dendritic trees [26]. In order to test if histamine treatment elicits similar responses from neurons in MBO slice cultures, immunostaining with antibodies specific for vimentin was performed on histological sections of MBO cultures with and without histamine treatment. As shown in Figure 3, histamine treatment is accompanied by increased density of neurons expressing vimentin (indicated by red arrows, Figure 3(b) versus Figure 3(a)) while vimentin expression is generally restricted to vascular endothelial cells in untreated samples. It is also noteworthy that staining for vimentin is much weaker in the vimentin-positive neurons from the controls compared to those from histamine treated sections. Images from these samples were quantitated and the result is plotted in Figure 3(c). Similar to GFAP, the source of the sections (proximal, middle, or distal) does not make a difference in the density of vimentin-positive neurons (untreated, *P* = 0.5495; treated, *P* = 0.5815; one-way ANOVA). However, compared to those untreated controls, a greater than fourfold increase is detected in the histamine-treated samples (Figure 3(c)). This conclusion holds true regardless of the proportion of the slices in the MBO cultures. Thus, we conclude that histamine treatment produces neuronal responses as measured by increased vimentin in MBO slice cultures (Figure 3).

### 3.4. Histamine Induced Changes in MBO Cultures Are Independent of the Slice's Thickness and Resemble the Pathology in AD Patients

**Figure 4:**
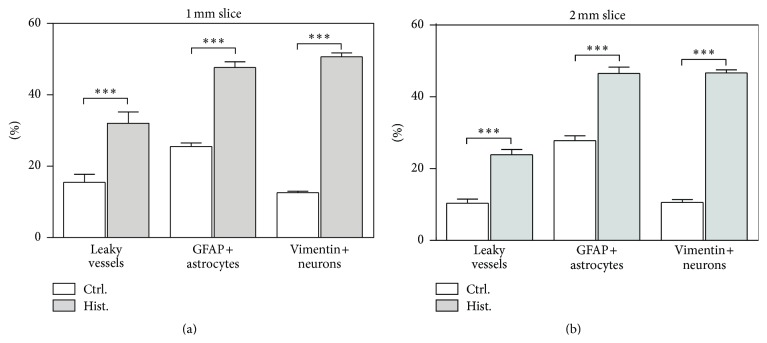
Histamine is able to induce pathological changes in MBO cultures, regardless of their thickness. Treatment of both 1 mm (a) and 2 mm (b) thick MBO cultures with histamine leads to an increase in blood vessel leakage, astrocyte activation, and neuronal damage responses, compared to controls. Mean ± SEM is used to represent the variations within each group. ^*∗*^ *P* < 0.05; ^*∗∗*^ *P* < 0.01; ^*∗∗∗*^ *P* < 0.001.

In order to determine if the thickness of the slices had a significant influence on the results, slices with 1 mm thickness were generated and used in the same experimental setting. The results are plotted in Figure 4(a). As a comparison, the results with 2 mm thickness are also presented (Figure 4(b)). The changes are comparable between the 1 mm slices and 2 mm ones among the three parameters tested. As described previously, overall the 2 mm MBO cultures display an increased BV leakage [(23.87 ± 1.46)%, histamine-treated versus (10.38 ± 1.16)%, control], elevated astrocyte activation [(46.51 ± 1.75)%, histamine-treated versus (27.83 ± 1.32)%, control], and a severe neuronal damage-response [(46.66 ± 0.87)%, histamine-treated versus (10.59 ± 0.79)%, control]. Within the 1 mm MBO cultures, similar effects are perceived, where (47.66 ± 1.64)% of GFAP-positive astrocytes are detected in the histamine-treated samples while only (25.54 ± 1.00)% are detected in the control; (50.68 ± 1.09)% of neurons display vimentin in the treated ones compared to (12.55 ± 0.41)% in the control; and the BV leakage increases from (15.49 ± 2.26)% to (32.00 ± 3.15)% upon histamine treatment. In general, there is about a twofold increase in percentage of leaky BVs and activated astrocytes, while there is a fourfold increase in the vimentin-positive neurons, irrespective of the slice thickness when the MBO slices are treated with histamine.

To compare the pathological changes in MBO cultures with those in AD patients, immunohistochemistry for IgG, GFAP, and vimentin was also performed of human brain sections. The representative images from sections from AD patients (panels B, D, and F in Supplementary Figure  1 in Supplementary Material available online at http://dx.doi.org/10.1155/2015/937148) as well as age-matched, neurologically normal brains that served as controls (panels A, C, and E in Figure S1) are presented. Breaches in BBB indicated by the efflux of IgG, astrocyte activation marked by increased GFAP expression, and a neuronal damage-response highlighted by increased vimentin expression are all observed. These observations are consistent with previous reports [7, 26] and most importantly validate the use of the MBO culture system in investigating AD.

## 4. Discussion

The results presented above demonstrate that* in vitro* histamine treatment of MBO cultures can induce BBB breaches (Figure 1), astrocyte activation (Figure 2), and neuronal damage response (Figure 3), all of which are observed in AD patients (Figure S1). These results also lead to the conclusion that histamine is able to permeate the brain interstitium within a short time and this effect is not influenced by the thickness of the slice tested (Figure 4).

**Figure 5:**
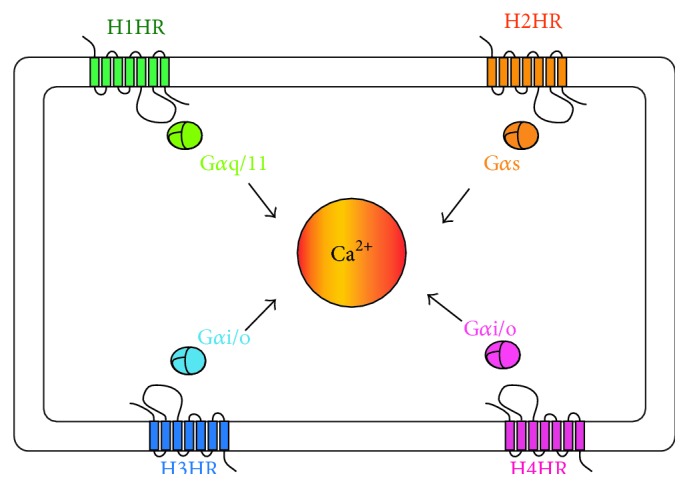
Histamine signaling through different receptor types converges at cellular calcium levels. The four major types of histamine receptors interact with different G*α* proteins as indicated. The subsequent steps in the signaling pathway are variable. However, one common feature is that they all affect cellular calcium levels.

Histamine is known to bind to four different receptors: H1R, H2R, H3R, and H4R. These histamine receptors are known to interact with different G proteins and, subsequently, activate different signaling cascades (Figure 5). However, one common thread among these is through calcium (Ca^2+^). To arrive at possible mechanisms by which histamine exerts its effects on the MBO culture system, the implications of current knowledge in each of the three major cell-types involved in this study—endothelial cells, glial cells, and neurons—are discussed below.

### 4.1. Histamine and BBB Breakdown: H2R on Endothelial Cells?

BBB breakdown is an important pathology commonly associated with AD, as it allows for the extravasation of potentially damaging humoral elements such as autoimmune antibodies, complement components, A*β*42, and proinflammatory mediators [7, 50–52]. It has also been shown that, in dynamic systems, histamine stresses the tight junctions between adjacent endothelial cells via histamine receptors, creating intercellular space that allows for the leakage of humeral elements into the surrounding parenchyma [53–55]. All of the four histamine receptors, H1R, H2R, H3R, and H4R, have been reported to be expressed on brain endothelial cells and playing critical roles in regulating BBB permeability (Table 1) [56, 57]. H1R signaling reduces BBB permeability [58], while H2R signaling elevates Ca^2+^ level and leads to increased BBB permeability [49]. The knockout mice for both H3R and H4R show greater BBB permeability compared to WT mice [59, 60]. In our current study with MBO cultures, we demonstrate that histamine increases the BBB permeability leading to extravasation of serum components as indicated by IgG leakage. Since that effect is associated with the H2R and H2R is expressed in endothelial cells, we propose that histamine, most likely, acts through H2R on the endothelial cells.

Histamine has been previously shown to cause significant increases in intracellular Ca^2+^ in endothelial cells [61, 62]. This elevated Ca^2+^ level causes cellular contraction and finally leads to an increase in the permeability of the BBB [16]. On the other hand, Ca^2+^ dysregulation also plays an important role in AD pathology, where it may lead to a variety of changes including cellular loss [63, 64]. Alteration in Ca^2+^ regulation has been reported in multiple mouse models of AD [63, 65]. Further experimental investigation is in progress to evaluate the role of H2R and determine if the mechanism of action on endothelial cells involves Ca^2+^.

### 4.2. Histamine-Induced Gliosis: Indicator of Inflammatory Damage in MBO Cultures

Inflammation within the brain parenchyma contributes significantly to AD pathogenesis (see [6] for an extensive review). An established and accepted marker of such inflammation is astrocyte activation, also known as gliosis, and observed through upregulation of GFAP [66]. Activated astrocytes are evident in the brain from transgenic animal models of AD [6, 67] as well as in the regions surrounding amyloid plaques from the AD patients [68, 69]. In the present study, we show that histamine is able to increase gliosis in MBO cultures by almost twofold.

Three of the four histamine receptors—H1R, H2R, and H3R—are all expressed in astrocytes, but no H4R has been reported (Table 1). A strong correlation between increase in the H3R, GFAP, and vimentin mRNA levels has been reported in postmortem AD brains [70]. Previous literature also demonstrates that H1Rs expressed on astrocytes regulate cytoplasmic Ca^2+^ via PKC-MAPK signaling pathway [71–73]. Therefore, the observed increase in GFAP expression here may be caused by a direct interaction between histamine and astrocytes via more than one receptor. It is conceivable that the effect may not be mediated by histamine receptors on the astrocytes. Since it is known that astrocytes respond to BBB breakdown by a swelling of their foot processes [38, 39], the possibility that the BBB breach induced by histamine causes the increase in astrocytic GFAP cannot be ruled out. In either case, histamine is responsible for the increased gliosis in the MBO cultures that resemble AD. Therefore, gliosis, a pathological change downstream to BBB breakdown, is effectively observable in the MBO cultures, attesting the power of the MBO system in recapitulating pathologies seen in AD patients and also in exploring the potential mechanisms underlying it.

### 4.3. Histamine and Neuron Damage Response: Primary or Secondary?

Vimentin is an intermediate filament protein that is important for neuronal growth/development and is necessary for the extension and branching of neurites [74]. Thus, it is commonly expressed by neuronal precursor cells in the developing CNS of both rodents and humans [24, 26, 75]. In the healthy, adult brain, vimentin expression is mainly restricted to endothelial cells [24, 74, 76, 77]. However, in the AD brain, vimentin has been found within neurofibrillary tangles, a pathological hallmark associated with the disease [78, 79]. Moreover, we have shown that vimentin is expressed by neurons in AD brains, possibly as part of a damage-response mechanism in order to reestablish their dendritic trees [26].

In our present study, histamine administration results in perinuclear vimentin expression within the cell bodies of neurons, whereas it is mainly restricted to the vascular epithelium of control MBO cultures. Neurons throughout the brain express histamine receptors, H1R, H2R, and H3R; however, there is no consensus on H4R (Table 1). Therefore, it is possible that histamine directly binds to neurons in MBO cultures, causing localized damage due to excitotoxicity and inducing the neuron's damage-response, including expression of vimentin. Increase in the H3R and vimentin has been reported in postmortem AD brains [70]. Further experiments are under investigation to determine the subtypes of histamine receptors on the responding neurons in the MBO cultures. Alternatively, the activation of vimentin expression could be secondary to the BBB breakdown caused by histamine, which could allow plasma components, such as brain-reactive autoantibodies, complement components, and A*β*42, to directly bind to and damage the MBO neurons, as has been shown in AD brains [7, 21–23]. Histamine's ability to elicit the neuronal response within such a short time period in MBO cultures shows the immediacy of the neuronal damage-response mechanism. Within the relatively brief 1 hour of histamine exposure in this study, neurons already begin to repair themselves and reestablish their lost connections so that they can continue to function normally. Moreover, the fact that so many neurons display a damage response in the present study indicates the overall extent to which histamine can mediate damage in the brain. For those reasons, it suggests that the MBO cultures have a significant potential to investigate neuronal repair mechanisms.

### 4.4. Histamine and AD

We have shown histamine to be a powerful molecule in terms of its abilities to create pathological changes in MBO cultures that are consistent with those found in AD. This is in line with its proinflammatory nature. Interestingly, it has been previously noted that the use of anti-inflammatory drugs has benefits in treating the cognitive symptoms of AD [80–86]. Yet, the merits of utilizing antihistamines as a treatment may largely depend on the timing of administrations since the cellular changes found in AD have been shown to predate the symptomology by years to decades [87].

It is entirely possible that histamine-induced inflammation is an early and/or downstream contributor in AD. After all, histamine is capable of creating several pathologies consistent with the disease process and triggering a cascade of problems. As shown in the present paper, histamine can cause the BBB breakdown that allows A*β*42 and other humoral element access to cells of the brain. Once histamine enters the brain parenchyma, it could potentate these adverse effects by damaging neurons, thus resulting in increases in gliosis.

Cells in MBO cultures are not only capable of being damaged by histamine, but also responding to that damage; that is, astrocytes respond to inflammatory damage by undergoing gliosis and neurons respond to damage by upregulating vimentin production. As such, our histamine-treated MBO model system provides a useful model to not only study the effects of inflammatory damage as seen in AD, but also to study the way in which the brain responds to this damage. In conclusion, our current study indicates that MBO cultures treated with histamine are a quick, simple, and effective tool for investigating pathological changes, some of which are demonstrably associated with AD.

## 5. Conclusions

In the present study we show that when administered in MBO cultures, histamine is able to induce BBB breaches and cellular pathologies that were observed in AD. Results suggest that histamine is a potent mediator of BBB breakdown, astrocyte activation, and neuronal damage response. Therefore, we propose that MBO cultures treated with histamine can provide a quick and powerful model system to study the cellular pathologies associated with AD and to test potential medications to reverse or mitigate these pathological changes.

## Supplementary Material

AD brains display BBB breach, astrocyte activation and neuronal damages.
To investigate the pathological hallmarks of AD, immunohistochemistry (IHC) for IgG, GFAP and vimentin was performed and representative images are presented. Immunostaining was carried out on sections from brains of AD patients (Fig. S1, Panels B, D and F) as well as age-matched, neurologically normal brains that served as controls (Fig. S1, Panels A, C and E). Firstly, in AD brains, extravasated IgG is localized to perivascular leak clouds emerging from a discrete region along the length of vessels (demarcated with a dotted outline; Fig. S1B), which demonstrates that AD patients display BBB breakdown. Conversely, perivascular leak clouds are rare and IgG is restricted to the lumen of blood vessels in control (Fig. S1A). Secondly, AD brains show a marked increase in the density of activated astrocytes (indicated by arrowheads; Fig. S1D), as determined by an increased intensity of immunostaining for GFAP compared to control brains (Fig. S1C). Thirdly, the cell bodies and apical dendrites of large pyramidal neurons within areas of pathology in AD brains are selectively vimentin-positive (Fig. S1F), whereas vimentin expression is generally restricted to vascular endothelial cells in control brains (Fig. S1E). Therefore, the results above demonstrate that in AD patients, but not in neurologically normal age-matched controls, breaches in BBB indicated by the efflux of IgG, astrocyte activation marked by increased GFAP expression and a neuronal damage-response highlighted by increased vimentin expression are all observed.
Supplementary Figure 1: (A) Control cortex (B) AD cortex shows BBB breakdown and extravasated IgG surrounding BV (dotted outline). (C) Control cortex with GFAP positive BVs (indicated by arrows) and few GFAP positive astrocytes (indicated by arrowheads). (D) AD cortex with more intensely GFAP positive astrocytes (indicated by arrowheads). (E) Vimentin expression is restricted to BVs (indicated by arrows) in control cortex and is negative for neurons (indicated by black arrow heads). (F) In AD cortex, vimentin is localized in the perikaryon and apical dendrite of pyramidal neurons (indicated by red arrow heads) as well as BV (indicated by arrow). All of the sections are visualized by DAB shown in brown and counterstained with hematoxylin to show nucleus in blue/purple as described in Section 2. (A-D) Scale Bar, 250 μm; (E-F) Scale Bar, 100 μm.

## Figures and Tables

**Table 1:** Multiple histamine receptor types are expressed in relevant cells and all affect BBB permeability.

Histamine receptor type	Expression in the cells relevant to the study	Effect on BBB permeability
H1R	Neurons [88–90], astrocytes [72, 91–93], endothelial cells [56, 57]	H1R signaling reduces BBB permeability [58]

H2R	Neurons [94], astrocytes [91, 92], endothelial cells [95]	H2R signaling increases BBB permeability [49]

H3R	Neurons [90, 96], astrocytes [92, 97], endothelial cells [56]	H3R knockout mice show greater BBB permeability compared to WT mice [59, 60]

H4R	Neurons? [98], endothelial cells [56, 98]	H4R knockout mice show greater BBB permeability compared to WT mice [59, 60]

The current knowledge on the expression of the histamine receptors in the three major cell-types relevant to the current study as well as the effect on BBB permeability is summarized. The citations are also provided.
