# Supplementary material for: Histamine Induces Alzheimer's Disease-Like Blood Brain Barrier Breach and Local Cellular Responses in Mouse Brain Organotypic Cultures
Source: Biomed Res Int. 2015 Nov 30;2015:937148. doi: 10.1155/2015/937148 (PMC4677161; doi:10.1155/2015/937148)
Supplement: Supplementary file 1 — AD brains display BBB breach, astrocyte activation and neuronal damages. To investigate the pathological hallmarks of AD, immunohistochemistry (IHC) for IgG, GFAP and vimentin was performed and representative images are presented. Immunostaining was carried out on sections from brains of AD patients (Fig. S1, Panels B, D and F) as well as age-matched, neurologically normal brains that served as controls (Fig. S1, Panels A, C and E). Firstly, in AD brains, extravasated IgG is localized to perivascular leak clouds emerging from a discrete region along the length of vessels (demarcated with a dotted outline; Fig. S1B), which demonstrates that AD patients display BBB breakdown. Conversely, perivascular leak clouds are rare and IgG is restricted to the lumen of blood vessels in control (Fig. S1A). Secondly, AD brains show a marked increase in the density of activated astrocytes (indicated by arrowheads; Fig. S1D), as determined by an increased intensity of immunostaining for GFAP compared to control brains (Fig. S1C). Thirdly, the cell bodies and apical dendrites of large pyramidal neurons within areas of pathology in AD brains are selectively vimentin-positive (Fig. S1F), whereas vimentin expression is generally restricted to vascular endothelial cells in control brains (Fig. S1E). Therefore, the results above demonstrate that in AD patients, but not in neurologically normal age-matched controls, breaches in BBB indicated by the efflux of IgG, astrocyte activation marked by increased GFAP expression and a neuronal damage-response highlighted by increased vimentin expression are all observed. Supplementary Figure 1: (A) Control cortex (B) AD cortex shows BBB breakdown and extravasated IgG surrounding BV (dotted outline). (C) Control cortex with GFAP positive BVs (indicated by arrows) and few GFAP positive astrocytes (indicated by arrowheads). (D) AD cortex with more intensely GFAP positive astrocytes (indicated by arrowheads). (E) Vimentin expression is restricted to BVs [file 937148.f1.pdf]

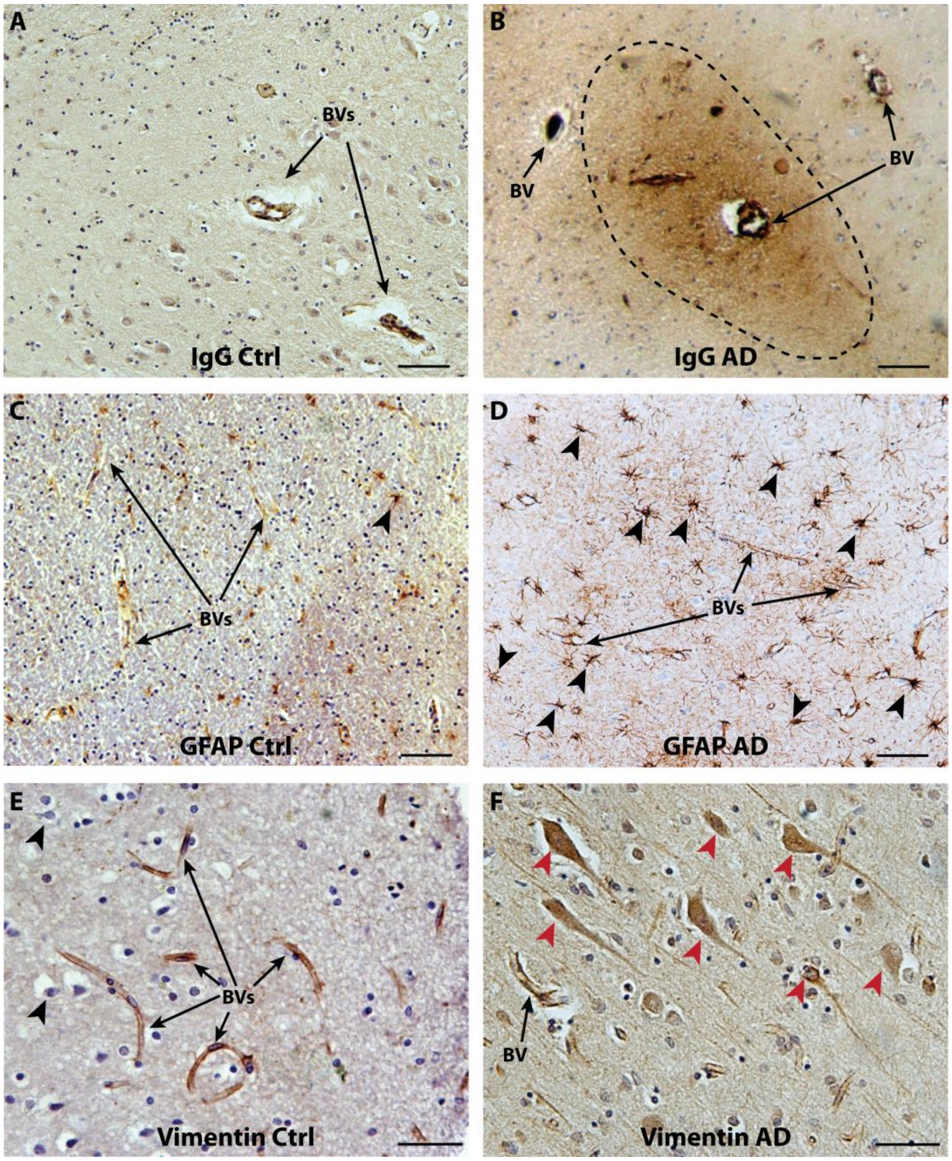

Supplementary Figure 1: Pathological changes in AD brains include breakdown of the BBB, activation of astrocytes, and neuronal expression of vimentin. (A) Control cortex shows IgG is confined to BV lumen. (B) Section of AD cortex shows BBB breakdown and extravasated IgG surrounding BV as demarcated by dotted outline. (C) Control cortex exhibits GFAP positive BVs (indicated by arrows) and few GFAP positive astrocytes (indicated by arrowheads). (D) AD cortex displays more intensely GFAP positive astrocytes (indicated by arrowheads). (E) Vimentin expression is restricted to BVs (indicated by arrows) in control cortex while it is negative for neurons (indicated by black arrow heads). (F) In AD cortex, vimentin is localized in the perikaryon and apical dendrite of pyramidal neurons (indicated by red arrow heads) as well as BV (indicated by arrow). All of the sections are visualized by DAB shown in brown and counterstained with hematoxylin to show nucleus in blue/purple as described in the Materials and Methods. (A-D) Scale Bar, 250 µm; (E-F) Scale Bar, 100 µm.
